# MyD88 Regulates the Expression of SMAD4 and the Iron Regulatory Hormone Hepcidin

**DOI:** 10.3389/fcell.2018.00105

**Published:** 2018-08-31

**Authors:** Macha Samba-Mondonga, Annie Calvé, Frédérick A. Mallette, Manuela M. Santos

**Affiliations:** ^1^Nutrition and Microbiome Laboratory, Centre de Recherche du Centre Hospitalier de l’Université de Montréal (CRCHUM), Montréal, QC, Canada; ^2^Département de Médecine, Université de Montréal, Montréal, QC, Canada; ^3^Centre de Recherche de l’Hôpital Maisonneuve-Rosemont, Université de Montréal, Montréal, QC, Canada

**Keywords:** MyD88, BMP6, SMAD4, iron, hepcidin, L265P mutation, SMAD6, Waldenström’s macroglobulinemia

## Abstract

The myeloid differentiation primary response gene 88 (MyD88) is an adaptive protein that is essential for the induction of inflammatory cytokines through almost all the Toll-like receptors (TLRs). TLRs recognize molecular patterns present in microorganisms called pathogen-associated molecular patterns. Therefore, MyD88 plays an important role in innate immunity since its activation triggers the first line of defense against microorganisms. Herein, we describe the first reported role of MyD88 in an interconnection between innate immunity and the iron-sensing pathway (BMP/SMAD4). We found that direct interaction of MyD88 with SMAD4 protein activated hepcidin expression. The iron regulatory hormone hepcidin is indispensable for the intestinal regulation of iron absorption and iron recycling by macrophages. We show that MyD88 induces hepcidin expression in a manner dependent on the proximal BMP responsive element on the hepcidin gene (*HAMP*) promoter. We identified the Toll/interleukin-1 receptor (TIR) domain of MyD88 as the domain of interaction with SMAD4. Furthermore, we show that BMP6 stimulation, which activates SMAD6 expression, also induces MyD88 proteosomal degradation as a negative feedback mechanism to limit hepcidin induction. Finally, we report that the MyD88 gain-of-function L265P mutation, frequently encountered in B-cell lymphomas such as Waldenström’s macroglobulinemia, enhances hepcidin expression and iron accumulation in B cell lines. Our results reveal a new potential role for MyD88 in the SMAD signaling pathway and iron homeostasis regulation.

## Introduction

The myeloid differentiation primary response gene 88 (MyD88) plays an important role in the mammalian host defense (Deguine and Barton, 2014). This universal adaptor protein is part of the family of signal transduction molecules required for the production of pro- or anti-inflammatory cytokines (Akira, 2003; Deguine and Barton, 2014) in response to IL-1R1 (Muzio et al., 1997; Burns et al., 1998) and Toll-like receptor (TLR) signaling (Medzhitov et al., 1998). TLR3 is the exception as it exclusively uses the adaptor molecule Toll/interleukin-1 receptor (TIR)-domain-containing adapter-inducing interferon-β (TRIF) (Yamamoto et al., 2003).

TLRs/MyD88 signaling has been shown to also contribute to the regulation of hepcidin, a molecule involved in cellular and systemic iron metabolism (Wang et al., 2009; Xiong et al., 2016; Lee et al., 2017). Hepcidin, encoded by the *HAMP* gene, is the major regulator of intestinal iron absorption and iron recycling from macrophages (Hentze et al., 2010). Hepcidin modulates cellular iron export by binding to Ferroportin 1, the only known cellular iron exporter in vertebrates, and inducing its degradation (Donovan et al., 2000; McKie et al., 2000). Since Ferroportin 1 is expressed on duodenal enterocytes absorbing dietary iron and on macrophages in liver and spleen, high hepcidin levels leads to the suppression of intestinal iron absorption and the accumulation of iron in macrophages (Rishi et al., 2015). Prolonged activation of hepcidin with consequent iron sequestration manifested by hypoferremia, may lead to the development of anemia of chronic diseases (ACD) or anemia of inflammation (Ganz and Nemeth, 2009). ACD is characterized by the presence of adequate iron stores, as defined by conventional criteria, but with insufficient iron mobilization from stores to appropriately support erythropoiesis. ACD is prevalent in patients suffering from infections, malignancies and auto-immune disorders, and it is linked with immune activation, exemplifying the interplay between iron metabolism and immune function (Weiss, 2009).

Besides being regulated by inflammatory stimuli though IL-6/STAT3 signaling (Wrighting and Andrews, 2006; Verga Falzacappa et al., 2007), hepcidin expression is also modulated though an iron-sensing pathway involving bone morphogenetic proteins (BMP), such as BMP6, and SMAD4 signaling (Ganz and Nemeth, 2012; Sheftel et al., 2012). BMPs are part of the transforming growth factor-β (TGF-β) superfamily of proteins, which includes TGF-βs and activins, among others. The iron signaling-pathway can initiate with BMP6, which is activated by increased iron stores (Kautz et al., 2011). BMP6 binds to the heteromeric complexes containing types I and II BMP receptors (BMPRI/II) (Parrow and Fleming, 2014), resulting in the recruitment and subsequent phosphorylation of SMADs 1, 5, and 8 (SMAD1/5/8) in the cytoplasm (Kautz et al., 2008). In turn, phosphorylated SMAD1/5/8 proteins (pSMAD1/5/8) form heteromeric complexes with SMAD4 that translocate into the nucleus to modulate the transcription of target genes, including *HAMP* (Casanovas et al., 2009). In addition to BMP6, other molecules such as BMP2 (Canali et al., 2017; Koch et al., 2017) and the peptide hormone Activin B have also been shown to induce hepcidin expression through SMAD1/5/8 signaling (Besson-Fournier et al., 2012; Canali et al., 2016).

As with other genes regulated through the TGF-β/BMP/SMAD signaling pathway, hepcidin is also regulated through a negative feedback loop by inhibitory SMADs, SMAD6 and SMAD7 (Mleczko-Sanecka et al., 2010; Vujic Spasic et al., 2013), which antagonize the activation of receptor-regulated SMADs. Inhibitory SMADs associate with activated TGF-β superfamily type I receptors, thereby preventing phosphorylation of receptor-regulated SMADs (Itoh and ten Dijke, 2007). SMAD7 inhibits both TGF-β/activin and BMP signaling, while SMAD6 efficiently inhibits BMP signaling but only weakly inhibits TGF-β/activin signaling (Hata et al., 1998; Ishisaki et al., 1999; Hanyu et al., 2001). The expression of inhibitory SMADs 6 and 7 is induced by activin/TGF-β and BMP signaling, thus creating a negative regulatory feedback loop (Imamura et al., 1997; Nakao et al., 1997).

Previously, we have shown that MyD88 plays an important role in the development of endotoxin-induced hypoferremia in mice (Layoun et al., 2012). More recently, we reported that *MyD88*^-/-^ mice are unable to appropriately regulate hepatic hepcidin levels in response to dietary iron overload (Layoun et al., 2018). This was associated with significantly reduced Smad4 protein levels in the livers of *MyD88*^-/-^ mice compared to wild-type mice. In the present study, we further investigated the link between MyD88, the SMAD/BMP signaling pathway, and hepcidin regulation.

## Materials and Methods

### Cell Culture and Treatments

MyD88 knockout (KO) human embryonic kidney 293 cells (HEK293-I3A) were a kind gift from G. Stark (Department of Molecular Genetics, Lerner Research Institute, Cleveland, OH, United States) (Li et al., 1999). HEK293-I3A cells were cultured in Dulbecco’s modified Eagle’s medium (DMEM; Wisent Inc., Montreal, QC, Canada). Huh7 human hepatoma cells (ATCC) were maintained in DMEM. HepG2 human hepatoma cells (ATCC) were maintained in Eagle’s minimal essential medium (EMEM; Wisent Inc.). Namalwa cells and Raji B cells were a kind gift from R. Bertrand and W. Mourad, respectively (Centre de Recherche du Centre Hospitalier de l’Université de Montréal, CRCHUM, Montréal, QC, Canada), and were cultured in Roswell Park Memorial Institute medium 1640 (RPMI1640; Wisent Inc.) Cell lines were supplemented with 10% fetal bovine serum (FBS; Wisent Inc.) and penicillin/streptomycin (Wisent Inc.), and were incubated at 37°C with 5% CO_2_. Cells were treated, where indicated, with 12.5, 25, and 50 ng/ml of either Activin B (R&D Systems, Minneapolis, MN, United States) or BMP6 (R&D Systems), or with 15 ng/ml of TGF-β (R&D Systems) for 24 hr. Huh7 cells were treated with 10 μM of the proteasome inhibitor MG132 (Sigma-Aldrich, St. Louis, MO, United States).

### Plasmids

Plasmid pCMV-HA-MyD88, also referred as pMyD88, contained full length MyD88 with hemagglutinin (HA) tag and was a gift from B. Beutler (Addgene plasmid #12287); pRK-DPC4-Flag contained SMAD4 with a Flag tag and was a gift from R. Derynck (Addgene plasmid #12627); pCI-His-hUbi contained ubiquitin with a histidine tag and was a gift from A. Winoto (Addgene plasmid #31815); pCS2-HA-SMAD6 contained SMAD6 with a HA tag (pSMAD6) and was a gift of J. Massague (Addgene plasmid #14962); and pRK-Myc-SMURF1 contained SMURF1 with a Myc tag (pSMURF1) and was a gift from Y. Zhang (Addgene plasmid #13676). All were purchased through Material Transfer Agreements (MTAs) with Addgene (Cambridge, MA, United States). Plasmid pCMV was created by removing MyD88 from pCMV-HA-MyD88 by digestion with restriction enzyme. The wild-type (-1234/+73) *HAMP* promoter reporter construct HAMP-Luc (*Firefly* luciferase) and *Renilla* luciferase reporter phRL-TK plasmid were used in the present study as previously described (Bagu and Santos, 2011). The QuickChange II site-directed mutagenesis kit (Agilent Technologies, ON, Canada) was used to generate the HAMP-LucΔBMP-RE1 construct, in which the BMP-RE1 site was mutated from GGCGCC to AGAACC (Verga Falzacappa et al., 2008). The site-directed mutagenesis kit was also used to create the pCMV-HA-MyD88 deletion mutants (ΔTIR, ΔDD, and ΔID). The glutathione *S*-transferase (GST)-SMAD4 fusion protein was produced by cloning SMAD4 from pRK-DPC4-Flag into pGEX-2TK (kind gift from I. Royal, CRCHUM). All constructs were confirmed by direct sequencing. Ready-made psiRNA Kit (ksirna42-hmyd88) containing psi-RNA-hMyD88 (psiRNA42-hMyd88-LucGl3), and the control plasmid psi-RNA-LucGL3 (psiRNA42-LucGl3) was purchased from InvivoGen (San Diego, CA, United States). psiRNA42-hMyd88LucGl3 sequences (F): AACUGGAACAGACAAACUAUCUCAA, (R):UUGACCUUGUCUGUUUGAUAGGAG. psiRNA42-LucGl3 sequences (F): GACUUACGCUGAGUACUUCGAUCAA, (R): UUCUGAAUGCGACUCAUGAAGCUGAG. The mutated form of the pCMV-HA-MyD88 plasmid, pCMV-HA-MyD88L265P, which results from a T to C mutation at position 794, was obtained using the QuickChange II site-directed mutagenesis kit (Agilent Technologies). The plasmid HAMP-MetLuc2 encoding a sequence-optimized, secreted luciferase was obtained by cloning the *HAMP* promoter reporter construct from HAMP-Luc into pMetLuc2-Reporter Vector (Clontech Laboratories, Mountain View, CA, United States). All plasmids were verified by digestion with restriction enzymes and sequencing (McGill University and Génome Québec Innovation Centre).

### Transfection and Co-immunoprecipitation Assays

MyD88-deficient HEK293-I3A and Huh7 cells were transiently transfected using Lipofectamine^TM^ 2000 (Invitrogen, Burlington, ON, Canada) as recommended by the manufacturer with indicated plasmids. The total amount of DNA was kept constant. For assessment of SMAD4 and MyD88 interactions, MyD88 KO HEK293-I3A cells were used. Twenty-four hours after transfection, cells were lysed in 1 mL RIPA buffer containing 50 mM Tris (pH 8), 150 mM NaCl, 1% NP40, 0.5% deoxycholic acid, 0.1% SDS, and protease inhibitor cocktail (Complete Mini, Roche, Mannheim, Germany). Cell lysates were then incubated with indicated antibodies (anti-Flag, anti-HA, or anti-MyD88 antibody) for 3 h at 4°C, after which EZview Red Protein A Affinity Gel (Sigma-Aldrich) was added for another 2 h. For control reactions, mouse IgG1 (Santa Cruz) was used. The immune complexes were precipitated and washed thoroughly with RIPA buffer. Immunoprecipitated proteins were then eluted by adding sample buffer and were subsequently fractionated by SDS-polyacrylamide gel electrophoresis (PAGE) and visualized by immunoblotting with anti-Flag and anti-HA antibodies. Lysates were also immunoblotted for expression of transfected SMAD4-Flag (pRK-DPC4-Flag) and HA-MyD88 (pCMV-HA-MyD88).

### si-RNA Transfection

Huh7 cells were transiently transfected with control psiRNA-LucGL3 (si-Ctrl) or psiRNA-hMyD88 (si-MyD88). Twenty-four hours after transfection, the cells were incubated with Zeocin at 400 μg/ml and BMP6 at 25 ng/ml for 24 h. Cell lysates were used for western blot analysis to verify the efficacy of protein knockdown by siRNA.

### Luciferase Reporter Assay

Huh7 cells were seeded at 1.1 × 10^5^ cell/ml onto 24-well plates. Cells were transiently co-transfected by lipofection using Lipofectamine^TM^ 2000 (Invitrogen). Lipofection included *Renilla* luciferase (phRL-TK) as the control reporter and the *Firefly* luciferase under the control of the *HAMP* promoter (HAMP-Luc) (Bagu and Santos, 2011; Bagu et al., 2013) in combination with pCMV-HA-MyD88 (pMyD88) or empty vector pCMV-HA (also referred as pCMV). The total amount of DNA was kept constant. After 24 h, cells were harvested and luciferase activity was measured by the Dual-Luciferase reporter assay system (Promega, Mississauga, ON, Canada). In all cases, the data were normalized for transfection efficiency by dividing *Firefly* luciferase activity by *Renilla* luciferase activity. Namalwa and Raji B cells were seeded at 1 × 10^6^ cell/ml and were transfected by electroporation with pCMV-HA, pCMV-HA-MyD88, or pCMV-HA-MyD88L265P along with HAMP-MetLuc2 using the Gene Pulser (Bio-Rad Laboratories, Mississauga, ON, Canada). After 24 h, cells were harvested and luciferase activity was measured by using the Ready-To-Glow Secreted Luciferase Reporter Assay (Clontech Laboratories) with a Victor3 1420 Multilabel Counter (Perkin Elmer life and Analytical Sciences, Turku, Finland). For long-term experiments, Huh7 cells were stably co-transfected with HAM-MetLuc2 along with pCMV or pMyD88 and were selected using G418 antibiotic (Invitrogen; 200 mg/ml), which was added in the culture medium 24 h post-transfection. Luciferase activity was measured at 24 h, 48 h, 2 weeks, 3 weeks, and 4 weeks after transfection.

### GST Pull-Down Assays

The GST-SMAD4 fusion protein was induced with 0.1 mM IPTG in BL21 (C2530H) *Escherichia coli* competent cells (New England BioLabs, NEB, MA, United States) ) transformed with pGEX-2TK-SMAD4 and after 4 h, the bacteria were lysed in 2% sarkosyl-STE buffer (10 mM Tris pH 8, 150 mM NaCl, 1 mM EDTA) and sonicated. HEK293-I3A cells were transfected with pCMV-HA-MyD88 or one of the three pCMV-HA-MyD88 deletion constructs. Total cellular lysate was extracted with RIPA buffer. Ten micrograms of GST-SMAD4 fusion protein or GST (as control) were incubated with Glutathione Sepharose 4B (GE Healthcare) for 1 h at 4°C. Beads were washed three times in TIF buffer (150 mM NaCl, 20 mM Tris pH 8, 1 mM MgCl2, 0.1% NP-40, and 10% glycerol), incubated with 50 μg of total cellular lysate for 1 h at 4°C, and washed again three times in TIF buffer. Pulled-down proteins were eluted by adding sample buffer, then fractionated by SDS-PAGE and visualized by immunoblotting with anti-HA and anti-GST antibodies. Lysates were also immunoblotted for expression of transfected HA-MyD88.

### SDS–PAGE and Western Blot Analysis

Cells were lysed in RIPA lysis buffer. Nuclear extracts were prepared with Nuclear Extract Kits (Active Motif, Carlsbad, CA, United States). Total cell lysates or nuclear and cytosol protein extracts were separated by 10% SDS–PAGE and blotted onto nitrocellulose membranes (Bio-Rad Laboratories). The membranes were immunoblotted with antibodies against the following: SMAD4 (1:500) (Santa Cruz Biotechnology, Santa Cruz, CA, United States), MyD88 (1:1000) (Cell Signaling, Danvers, MA, United States), phosphorylated SMAD5 (1:1000) (Abcam, Cambridge, MA, United States), SMAD1 (1:1000) (Cell Signaling), His (1:1000) (Genscript, Piscataway, NJ, United States), Flag (1:5000) (Genscript), HA (1:5000) (Genscript), Ubiquitin FK2 (1:100), Ferroportin 1 (1:1000) (Novus Biologicals, Littleton, CO, United States), GST (1:1000) (Genscript), and β-actin (1:10000) (Abcam, Cambridge, MA, United States). For secondary antibodies, anti–rabbit IgG (1:5000) or anti–mouse IgG (1:5000) were used. Antigen-antibody complexes were visualized with the ECL Western Blotting Detection Reagent (Invitrogen).

### Ubiquitination Assays

Huh7 cells were co-transfected with His-Ubi (pCI-His-hUbi) and HA-MyD88 or the empty vector pCMV-HA. After 24 h, cells were treated with Activin B and BMP6 at 25 ng/ml for 6 hr. Immunoprecipitation was performed using anti-His antibody (Genscript). For endogenous ubiquitin assay, Huh7 and HepG2 cells were transfected with HA-tagged MyD88 plasmid alone. After 24 h, cells were treated with Activin B and BMP6 at 25 ng/ml for 6 h. Immunoprecipitation was performed using anti- ubiquitin (FK2) antibody (EMD Millipore).

### Intracellular Iron Concentration

Namalwa and Raji B cells were lysed in 1 mL RIPA buffer, and iron concentration in lysates was determined using the QuantiChrom^TM^ Iron Assay Kit (Bioassay Systems, Hayward, CA, United States). The number of live/dead cells was determined by flow cytometry using propidium iodide dye (Invitrogen).

### Quantitative Reverse Transcription-Polymerase Chain Reaction (qRT-PCR)

Total RNA was isolated with Trizol reagent (Invitrogen), and reverse transcription was performed with the Omniscript RT kit (QIAGEN, Mississauga, ON, Canada). mRNA expression levels were measured by real-time PCR in a Rotor Gene 3000 Real Time DNA Detection System (Montreal Biotech, Kirkland, QC, Canada) with QuantiTect SYBR Green I PCR kits (QIAGEN) as described (Makui et al., 2005). The following primers were used: hepcidin - (F) CTCTGCAAGTTGTCCCGTCT and (R) ACCAGAGCAAGCTCAAGACC; β-Actin - (F) AGAAAATCTGGCACCACACC and (R) AGAGGCGTACAGGGATAGCA.

### Statistical Analysis

All statistics were calculated with Prism software (GraphPad, San Diego, CA, United States), with a pre-specified significant *P*-value of 0.05. Student’s *t*-test was performed or multiple comparisons were evaluated statistically by one-way analysis of variance (ANOVA), followed by the Bonferroni multiple comparison test.

## Results

### MyD88 Expression Influences SMAD4 Levels in Hepatoma Cells

**FIGURE 1:**
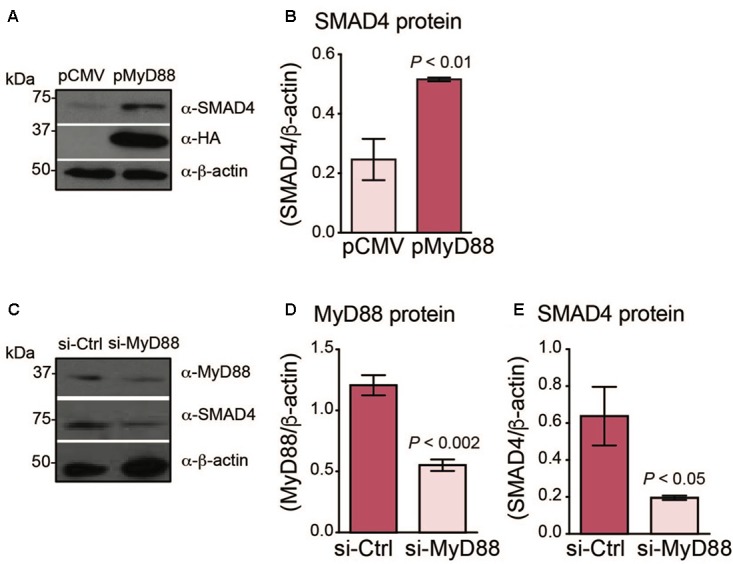
MyD88 expression levels regulate endogenous SMAD4 expression in Huh7 hepatoma cells. **(A,B)** MyD88 overexpression enhances endogenous SMAD4 expression. **(A)** Huh7 cells transiently transfected with an empty vector (pCMV) or HA-tagged MyD88 plasmid (pMyD88). Total cell lysates were analyzed by western blotting for endogenous SMAD4 expression. Expression of the β-actin protein was used as a loading control. **(B)** Densitometric quantification of SMAD4 levels in western blots from three independent experiments. **(C–E)** MyD88 repression lowers endogenous SMAD4 expression. **(C)** Representative western blot of MyD88 and SMAD4 expression in Huh7 cells transiently transfected with control psiRNA-LucGL3 (si-Ctrl) or psiRNA-hMyD88 (si-MyD88). Expression of the β-actin protein was used as a loading control. **(D,E)** Densitometric quantification of **(D)** MyD88 and **(E)** SMAD4 protein levels from three independent experiments. Results are presented as mean ± SEM. Statistical analyses were performed with Student’s *t*-test.

We previously found that Smad4 protein levels were diminished in the livers of dietary iron-loaded *MyD88*^-/-^ mice (Layoun et al., 2018). Therefore, we tested whether an *in vitro* system of hepatoma cells could demonstrate a similar association between MyD88 levels and SMAD4/hepcidin expression. We transfected Huh7 and HepG2 cells with HA-tagged MyD88 (pMyD88) or with empty plasmid (pCMV) and assessed the levels of endogenous SMAD4 protein. As shown in **Figures 1A,B** and **Supplementary Figures S1A,B**, **S2A,B**, SMAD4 levels increased when MyD88 was overexpressed. The SMAD4 pathway modulating hepcidin expression involves the phosphorylation of SMAD proteins 1, 5, and 8 (Kersten et al., 2005), which translocate into the nucleus where they regulate the transcription of hepcidin (Pantopoulos et al., 2012). Hence, we next analyzed Smad5 phosphorylation levels in pMyD88 transfected Huh7 cells. As shown in **Supplementary Figure S3**, MyD88 overexpression did not influence SMAD5 phosphorylation.

Next, we investigated whether the repression of MyD88 expression in Huh7 cells would result in a reduction of endogenous SMAD4. We generated a knockdown of endogenous MyD88 in the Huh7 cell line using the si-RNA MyD88 plasmid (si-MyD88) and compared with the control, scrambled si-RNA (si-Ctrl) (**Figures 1C,D**). Consistently, MyD88 knockdown resulted in a reduction of endogenous SMAD4 protein expression (**Figures 1C–E**).

### MyD88 Expression Levels Modulate Hepcidin Expression in Hepatoma Cells

**FIGURE 2:**
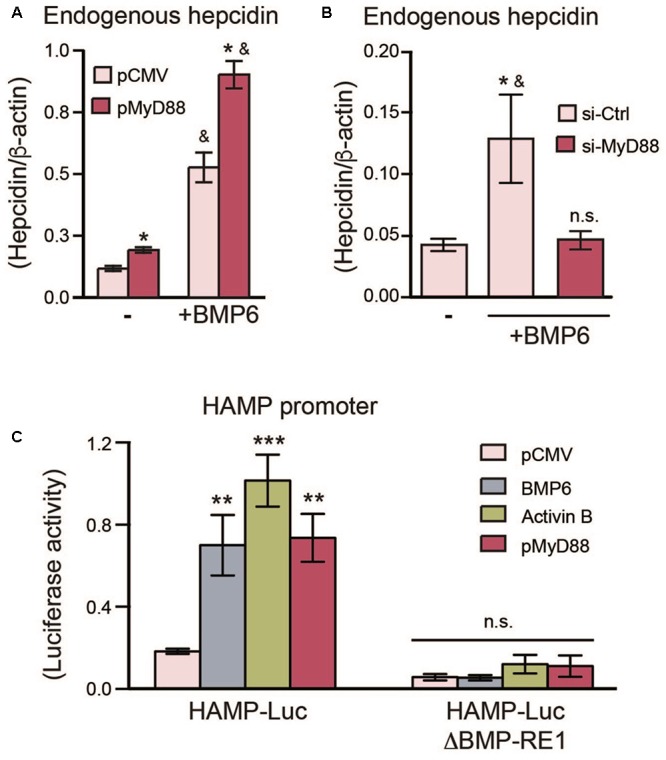
MyD88 expression levels regulate endogenous hepcidin expression in Huh7 cells. **(A)** MyD88 overexpression enhances endogenous hepcidin expression. Huh7 cells were transiently transfected with an empty vector (pCMV) or HA-tagged MyD88 plasmid (pMyD88) and treated with (+) or without (–) BMP6. Hepcidin (*HAMP*) mRNA levels were assessed by RT-PCR. Results are presented as mean ± SEM. ^∗^*P* < 0.01 compared with vector (pCMV) transfected cells and ^&^*P* < 0.01 compared to cells without BMP6 treatment. **(B)** MyD88 knockdown lowers endogenous hepcidin expression in Huh7 cells. Hepcidin mRNA levels in Huh7 cells were transfected with si-Ctrl or si-MyD88 and treated with BMP6 (+). Results are presented as mean ± SEM. ^∗^*P* < 0.05 compared with si-Ctrl transfected cells and ^&^*P* < 0.05 compared to cells transfected with si-MyD88 and treated with BMP6; n.s., not significant compared to untreated si-Ctrl transfected cells. **(C)** Mutation of the BMP-RE1 in the HAMP promoter abolishes HAMP-Luc induction by MyD88. Huh7 cells were transiently co-transfected with HAMP-Luc or mutated HAMP-LucΔBMP-RE1 along with phRL-TK (*Renilla* Luciferase) as an internal control, and MyD88 plasmids (pCMV or lMyD88). BMP6 and Activin B treatments were used as controls. Luciferase activity was assessed 24 h after transfection. Results are presented as mean ± SEM of the relative activity (*Firefly*/*Renilla* ratio). ^∗∗^*P* < 0.001, ^∗∗∗^*P* < 0.0001, and n.s., not significant compared with empty plasmid (pCMV). The results are representative of at least three independent experiments. Statistical analysis was performed with one-way ANOVA.

To further understand the link between MyD88 expression levels and hepcidin regulation, we measured endogenous levels of hepcidin in cells overexpressing MyD88. As shown in **Figure 2A** and **Supplementary Figure S1C**, MyD88 overexpression resulted in an increase of endogenous hepcidin mRNA levels, and this increase was further enhanced with the addition of BMP6, a signaling molecule activated by dietary iron-loading *in vivo* (Andriopoulos et al., 2009). Conversely, repression of MyD88 resulted in the abolishment of endogenous hepcidin mRNA induction by BMP6 (**Figure 2B**).

Next, we examined the effects of MyD88 on the SMAD4-dependent activation of hepcidin with a luciferase reporter-gene controlled by the human *HAMP* promoter (HAMP-Luc) (Bagu and Santos, 2011). In line with previous studies (Bagu and Santos, 2011; Besson-Fournier et al., 2012), both BMP6 and Activin B, two distinct BMP-signaling activators relevant to hepcidin induction, were able to activate the HAMP-Luc reporter gene (**Figure 2C** and **Supplementary Figure S1D**). In turn, MyD88 transfection also led to HAMP-Luc activation (**Figure 2C** and **Supplementary Figures S2C,D**). To further establish a link between MyD88 and the BMP-signaling pathway for hepcidin induction, we mutated the BMP-responsive element (BMP-RE1) located at position -84/-79 of the *HAMP* promoter (HAMP-LucΔBMP-RE1), which is essential for BMP6-mediated hepcidin activation. The mutation of this vital responsive element abolished the activation of *HAMP* promoter by MyD88 (**Figure 2C** and **Supplementary Figure S1D**) and, as expected by BMP6 and Activin B (Verga Falzacappa et al., 2008; Besson-Fournier et al., 2012).

Taken together, data in **Figures 1**, **2** show that MyD88 expression levels affect SMAD4 protein levels in Huh7 hepatoma cells, as well as hepcidin activation induced by BMP6 and Activin B through BMP-RE elements located on the *HAMP* promoter.

### MyD88 Directly Interacts With SMAD4 Through the Toll/Interleukin-1 Receptor (TIR) Domain of MyD88 Affecting Hepcidin Expression

**FIGURE 3:**
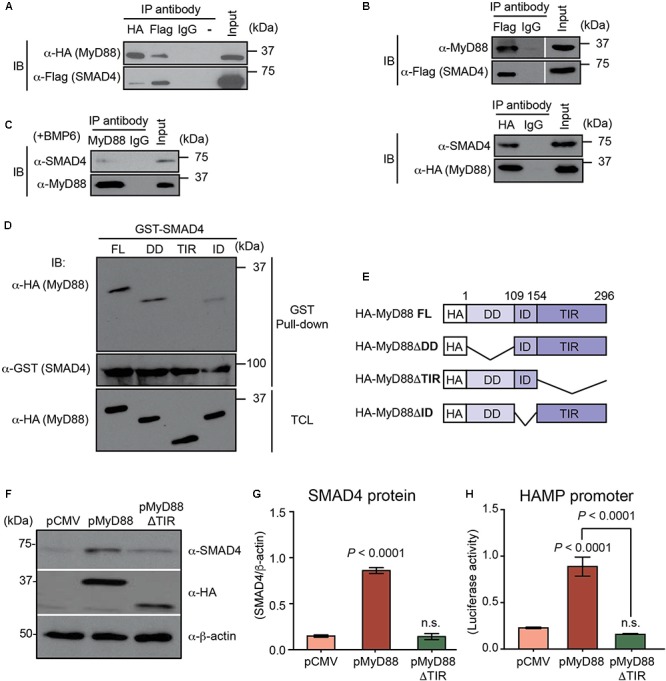
MyD88 directly interacts with SMAD4 through the Toll/Interleukin-1 receptor (TIR) domain of MyD88. **(A–C)** Co-immunoprecipitation of MyD88 with SMAD4. **(A)** MyD88 KO HEK293 cells (HEK293-I3A) were transfected transiently with HA-tagged MyD88 together with Flag-tagged SMAD4. Cell lysates were subjected to immunoprecipitation (IP) with the anti-Flag, anti-HA or normal IgG antibody (as a control) and analyzed by immunoblotting (IB) with an anti-HA antibody to detect MyD88 and an anti-Flag antibody to detect SMAD4. **(B)** Co-immunoprecipitation of endogenous MyD88 with Flag-tagged SMAD4 and, reciprocally, of endogenous SMAD4 with HA-tagged MyD88. **(C)** Co-immunoprecipitation of endogenous MyD88 with endogenous SMAD4 in Huh7 cells treated with BMP6 (+BMP6) for 24 h. **(D,E)** The TIR domain of MyD88 is necessary for its interaction with SMAD4. HEK293-I3A cells were transiently transfected with pCMV-HA-MyD88 or one of the three MyD88-deletion plasmids as shown in **(E)**. **(D)** Total cellular lysates (TCL) were extracted, incubated with GST-SMAD4, and analyzed by western blot with the indicated antibodies. **(E)** Schematic representation of plasmids encoding different truncated forms of MyD88. FL, full length; DD, death domain; TIR, Toll/Interleukin-1 receptor domain; ID, intermediate domain. **(F–H)** Defective MyD88 mutant (ΔTIR domain) abolishes the induction of endogenous SMAD4 and HAMP promoter activation by MyD88 overexpression. Huh7 cells were transfected with HAMP-Luc and pCMV or HA-tagged MyD88 vector (pMyD88) or the MyD88 vector lacking the TIR domain (pMyD88ΔTIR). **(F)** Expression of endogenous SMAD4 and transfected HA-tagged MyD88 was analyzed by western blotting. β-actin protein was used as a loading control. **(G)** Densitometric quantification of SMAD4 levels in western blots from three independent experiments. **(H)** Luciferase activity assessed 24 h after transfection. Results are presented as mean ± SEM of the relative activity (*Firefly*/*Renilla* ratio). Data are representative of a minimum of three experiments. Statistical analysis was performed with one-way ANOVA; n.s., not significant compared to pCMV.

Next, we hypothesized that MyD88 may influence SMAD4 levels by physically interacting with SMAD4. We transiently transfected Flag-tagged SMAD4 and HA-tagged MyD88 (HA-MyD88) expression vectors in the MyD88 KO cell line HEK293-I3A (Li et al., 1999), and performed immunoprecipitations. As shown in **Figure 3A**, HA-MyD88 co-immunoprecipitated with Flag-tagged SMAD4, and reciprocally, Flag-tagged SMAD4 co-immunoprecipitated with HA-MyD88, showing that SMAD4 can associate with MyD88. The SMAD4-MyD88 interaction was further confirmed by co-immunoprecipitating endogenous MyD88 with Flag-tagged SMAD4 and, reciprocally, endogenous SMAD4 with HA-tagged MyD88 in Huh7 cells (**Figure 3B**). Co-immunoprecipitations also detected interactions between endogenous SMAD4 and endogenous MyD88 in both Huh7 and HepG2 cells (**Figure 3C** and **Supplementary Figure S1H**).

To identify the regions of MyD88 that bind to SMAD4, the MyD88 protein was divided into three regions (**Figures 3D,E**) that have been previously described: the death domain (DD) at its N terminus; a C-terminal TIR domain required for TLRs and IL-1 receptor interaction; and a short connecting linker or intermediate domain (ID) (Bonnert et al., 1997; Beutler, 2009). We generated HA-tagged MyD88 deletion mutants specifically lacking one of these domains and then tested the ability of the MyD88 deletion mutants to directly interact with SMAD4 using GST pull-down assays. For this, we constructed a GST-fusion protein for SMAD4 (GST-SMAD4). As shown in **Figure 3D**, the overexpressed HA-MyD88 lacking the TIR domain (HA-MyD88ΔTIR) was the only deletion mutant that could not be pulled-down with GST-SMAD4, thus demonstrating that MyD88-SMAD4 interaction was mediated by the TIR domain of the MyD88 protein.

Since MyD88-SMAD4 interaction was mediated by the TIR domain of the MyD88 protein, we further analyzed the functional role of the MyD88 TIR domain in the BMP/SMAD4 pathway in hepatocytes. We transfected Huh7 cells with pMyD88 or the HA-tagged MyD88 deletion mutant lacking ΔTIR domain (pMyD88ΔTIR) and analyzed the expression of endogenous SMAD4 protein. Compared to wild-type MyD88, the MyD88ΔTIR mutant failed to upregulate SMAD4 protein levels (**Figures 3F,G**). In addition, we found that the defective ΔTIR domain MyD88 mutant also failed to activate the *HAMP* promoter when compared to wild-type MyD88, as assessed by luciferase assay (**Figure 3H**). Similar results using the MyD88ΔTIR mutant were obtained in HepG2 cells (**Supplementary Figures S1E–G**).

These results show that the TIR domain of the MyD88 adapter protein is essential for its role in modulating SMAD4 and hepcidin expression levels.

### MyD88 Regulation Through BMP6 and Activin B-Induced Degradation

**FIGURE 4:**
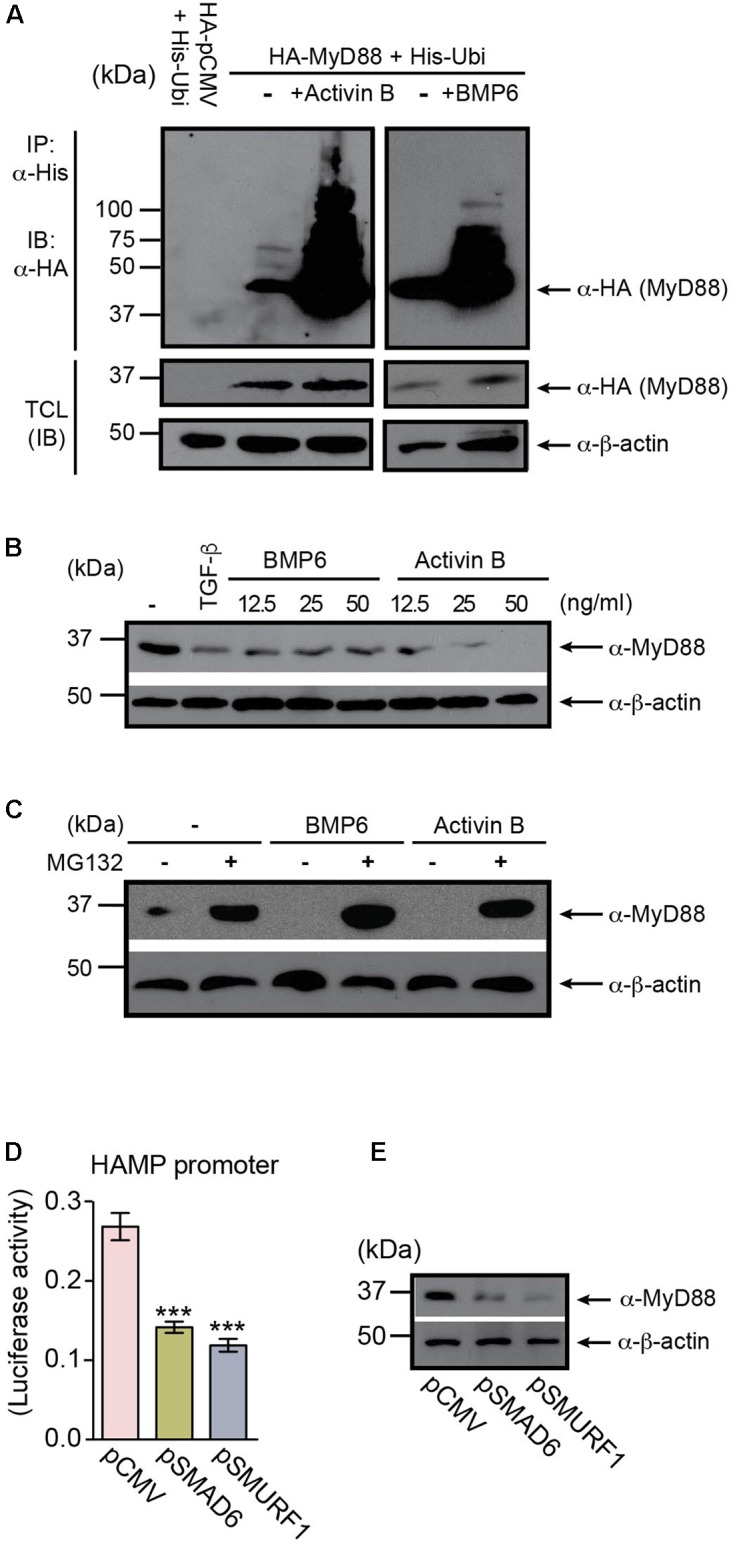
MyD88 is regulated through BMP6- and Activin B-induced degradation. **(A)** BMP6 and Activin B induce MyD88 ubiquitination. Huh7 cells were co-transfected with HA-tagged MyD88 and His-tagged Ubiquitin (His-Ubi), and treated with BMP6 or Activin B. HA-MyD88 ubiquitination was examined by immunoprecipitation (IP) using anti-His antibody, followed by immunoblotting (IB) with anti-HA antibody. Total cell lysates (TCL) before IP were immunoblotted with anti-HA and anti-β-actin antibodies. **(B)** BMP6 and Activin B reduce endogenous MyD88 protein levels. Huh7 cells were left untreated (-) or were treated (+) with BMP6 or Activin B at the specified doses (12.5, 25, and 50 ng/ml). TGF-β was used as a control (15 ng/ml). TCL were analyzed by immunoblotting using an anti-MyD88 antibody. Expression of the β-actin protein was used as a loading control. **(C)** The proteasome inhibitor MG132 prevents the degradation of MyD88 induced by BMP6 and Activin B. Huh7 cells were treated with BMP6 or Activin B (25 ng/ml) in the presence (-) or absence (-) of the proteasome inhibitor MG132 (10 μM) for 4 h. **(D,E)** Overexpression of SMAD6 and SMURF1 in Huh7 cells inhibits HAMP-Luc activity and correlates with changes in endogenous MyD88 expression. **(D)** Huh7 cells were transiently co-transfected with HAMP-Luc in combination with empty plasmid (pCMV), pSMAD6, or pSMURF1. Luciferase activity was assessed 24 h after transfection. Results are presented as mean ± SEM of the relative activity (*Firefly*/*Renilla* ratio). Statistical analysis was performed with one-way ANOVA. ^∗∗∗^*P* < 0.0001. **(E)** TCL were analyzed by immunoblotting using an anti-MyD88 antibody. Expression of the β-actin protein was used as a loading control. All data are representative of at least three independent experiments.

The BMP/SMAD signaling pathway is regulated by a negative feedback loop involving the inhibitory SMADs, particularly SMAD6 (Goto et al., 2007), which is upregulated in response to dietary iron-loading (Kautz et al., 2011). Previous studies have shown that in macrophages, SMAD6 negatively regulates MyD88 through degradation driven by the SMAD6-SMURFs (SMAD ubiquitin regulator factor proteins) pathway (Lee et al., 2011). We hypothesized that MyD88 levels may also be regulated by a negative feedback loop in hepatocytes involving BMP6 and Activin B signaling. Therefore, we assessed the ability of BMP6 and Activin B to trigger MyD88 degradation in Huh7 cells. First we examined the ubiquitination and degradation of HA-MyD88 in cells that were co-transfected with HA-MyD88 and histidine-tagged ubiquitin (His-Ubi). Both BMP6 and Activin B treatments induced MyD88 ubiquitination and degradation (**Figure 4A** and **Supplementary Figures S4**, **S5**). Accordingly, endogenous MyD88 levels in Huh7 cells were reduced by BMP6 and Activin B treatments (**Figure 4B**), and MyD88 degradation was inhibited by the proteasomal inhibitor MG132 (**Figure 4C**). We also show that overexpression of SMAD6 and SMURF1 in hepatoma cells inhibited HAMP-Luc activity (**Figure 4D** and **Supplementary Figure S1I**), while endogenous MyD88 protein expression diminished (**Figure 4E** and **Supplementary Figure S1J**). Furthermore, HAMP-Luc activity that was inhibited by SMAD6 and SMURF1 overexpression could be rescued by treatment with MG132 (**Supplementary Figure S6**).

These results indicate that signaling initiated by BMP6 and Activin B regulates MyD88 through a negative feedback loop relevant to the modulation of hepcidin expression in hepatocytes.

### The L265P Mutation of MyD88 Results in Enhanced Hepcidin Production

**FIGURE 5:**
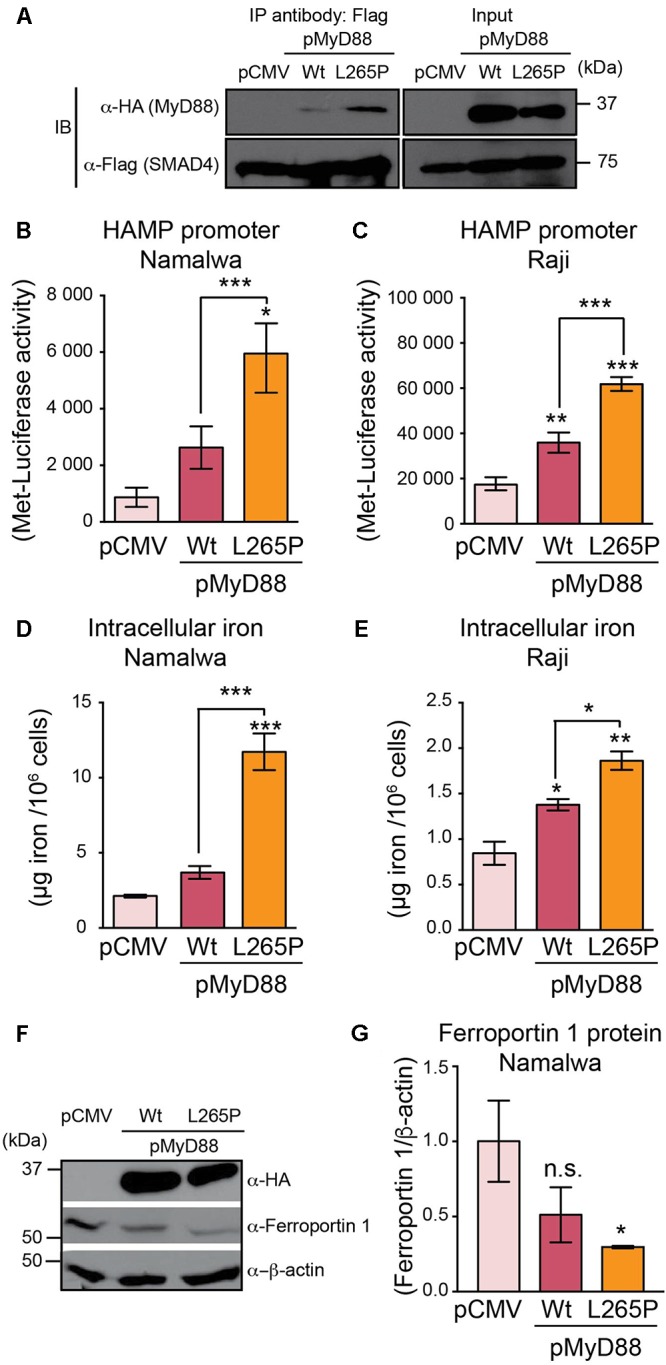
MyD88 L265P mutation enhances hepcidin expression and intracellular iron accumulation in B cell lines. **(A)** MyD88 L265P mutant interacts with SMAD4. MyD88 KO HEK293T cells (HEK293-I3A) were transfected transiently with either pCMV (control) or HA-tagged MyD88 (as Wt) or HA-MyD88L265P (as L265P), together with Flag-tagged SMAD4. Cell lysates were subjected to immunoprecipitation (IP) with the anti-Flag antibody and analyzed by immunoblotting (IB) with an anti-HA antibody to detect MyD88 and an anti-Flag antibody to detect SMAD4. Total cell lysates (Input) before IP were immunoblotted with anti-HA, anti-Flag, and anti-β-actin antibodies. **(B,C)** MyD88 L265P mutant upregulates hepcidin expression in B cell lines. **(B)** Namalwa and **(C)** Raji B cell lines were co-transfected with HAMP-MetLuc2 and control (pCMV) or MyD88 plasmids (pMyD88): wild-type (Wt) or mutated MyD88 (L265P). Luciferase activity was assessed 24 hr after transfection. Data are from a minimum of three independent experiments. **(D,E)** MyD88 L265P mutant increases intracellular iron content in B cell lines. **(D)** Namalwa and **(E)** Raji B cell lines were co-transfected with HAMP-MetLuc2 and control (pCMV) or MyD88 plasmids (pMyD88): wild-type (Wt) or mutated MyD88 (L265P). Intracellular iron concentrations were determined using the QuantiChrom Iron Assay Kit. Concentrations of iron are given in μg iron per 10^6^ live cells and data are from a minimum of three independent experiments. **(F,G)** MyD88 L265P mutant decreases Ferroportin 1 expression. **(F)** Namalwa cells were co-transfected with HAMP-MetLuc2 and control (pCMV) or MyD88 plasmids (pMyD88): wild-type (Wt) or mutated MyD88 (L265P). Total cell lysates were analyzed by western blotting for Ferroportin 1 and β-actin as loading control. **(G)** Densitometric quantification of Ferroportin 1 levels in western blots from three independent experiments. Statistical analysis was performed with one-way ANOVA. Results are presented as mean ± SEM. ^∗^*P* < 0.05, ^∗∗^*P* < 0.001, ^∗∗∗^*P* < 0.0001, and n.s., not significant compared with empty plasmid (pCMV).

In humans, genomic MyD88 mutations are extremely rare (Picard et al., 2011). In contrast, significantly higher rates of somatic MyD88 mutations have been identified in a variety of mature B cell tumors, with the most prevalent mutation being the Leu265Pro (L265P) missense substitution (Rossi, 2014). Most MyD88 mutations, including the L265P mutation in B cell tumors, cluster in the TIR domain, thus coinciding with the domain that we have identified as essential for interaction with SMAD4. Hence, we questioned whether the L265P mutation could affect hepcidin production in B cell lines. We generated an HA-MyD88 construct carrying the L265P mutation and used immunoprecipitation assays to show that the MyD88 L265P mutant could still interact and bind to SMAD4, similar to wild-type MyD88 (**Figure 5A**). We then co-transfected the wild-type MyD88 or MyD88 L265P mutant with HAMP-MetLuc2 in two B cell lines (Namalwa and Raji) and found that *HAMP*-driven luciferase activity was significantly higher in cells expressing the MyD88 L265P mutant compared to the wild-type (**Figures 5B,C**), indicating a gain-of-function of the L265P mutation in regards to hepcidin activation. When these experiments were repeated in Huh7 cells and the MyD88 KO cell line HEK293-I3A, we observed a similar enhanced induction of hepcidin by the MyD88 L265P mutant (**Supplementary Figure S7**).

In macrophages, hepcidin has been shown to regulate intracellular iron levels in an autocrine manner (Theurl et al., 2008). We reasoned that hepcidin produced in B cells may similarly regulate intracellular iron levels. Therefore, we measured the iron levels in the Namalwa and Raji B cell lines transfected with wild-type MyD88 or the MyD88 L265P mutant. As shown in **Figures 5D,E**, intracellular iron levels were significantly higher in B cell lines transfected with the MyD88 L265P mutant compared to wild-type MyD88 or the control pCMV vector, suggesting that this mutation of MyD88 results in enhanced accumulation of iron in B cells. In addition, we found that Ferroportin1 protein expression was significantly lower in Namalwa B cells transfected with MyD88 L265P mutant (**Figures 5F,G**), which is consistent with hepcidin modulating Ferroportin 1 expression through its internalization and degradation (Nemeth et al., 2004) resulting in enhanced intracellular iron accumulation.

Taken together these results indicate that the MyD88 L265P mutation leads to higher hepcidin expression and enhanced iron accumulation in B cells.

## Discussion

In previous studies, we found that *MyD88*^-/-^ mice cannot appropriately regulate hepcidin activation in response to iron-loading and that Smad4 expression is decreased in the nuclear extracts of these mice (Layoun et al., 2018). Here, we further investigate the involvement of MyD88 in the regulation of SMAD4 and hepcidin expression.

We show that MyD88 overexpression in Huh7 hepatoma cells increases endogenous SMAD4 protein levels, while conversely, suppression of MyD88 results in lowering SMAD4 levels. SMAD4 is pivotal to hepcidin regulation *in vivo*, as evidenced by mice with liver-specific Smad4 disruption that express very low levels of hepcidin in the liver, causing severe iron overload in several organs (Wang et al., 2005). Furthermore, transcriptional activation of hepcidin in response to known strong hepcidin stimulators such as iron, BMP and IL-6 in SMAD4-deficient hepatocytes from these mice is completely abrogated (Wang et al., 2005). Here, we show that MyD88-modulated SMAD4 expression in Huh7 also affects hepcidin expression: MyD88 overexpression induced hepcidin while repression of MyD88 inhibited hepcidin mRNA expression. Furthermore, MyD88 overexpression upregulated hepcidin promoter activity and, importantly, this was abolished in the absence of a functional BMP-responsive site on the HAMP promoter. These results show that, in our *in vitro* system, MyD88 modulates SMAD4 and consequent hepcidin expression through the BMP/SMAD4 signaling pathway.

We hypothesized that MyD88 could regulate SMAD4 levels through direct binding to SMAD4. Using co-immunoprecipitation assays, we show that, indeed, SMAD4 physically interacts with MyD88 protein. The physical interaction between SMAD4 and MyD88 protein could potentially be essential for translocation to the nucleus to prevent SMAD4 degradation or may facilitate SMAD4 interaction with the hepcidin promoter. We further identified the TIR domain of the MyD88 protein as the region involved in this interaction. Accordingly, the deletion of the TIR domain not only abolished the MyD88-SMAD4 interaction, but also MyD88-mediated stimulation of SMAD4 expression and hepcidin promoter activity. TIR domains of adapter proteins are known to assemble signaling components to trigger activation of transcription factors such as NF-κB and AP-1, as well as the overexpression of genes involved in the immune response (Bonnert et al., 1997; Ohnishi et al., 2009). Ultimately, interactions between adaptor proteins containing TIR domains activate transcription factors that regulate the expression of various proinflammatory cytokines (IL-1, IL-6, IL-8, and TNF-α) and chemokines (Narayanan and Park, 2015).

In addition to its role in innate immunity, the TIR domain of MyD88 directly interacts with SMAD6 (Lee et al., 2011) to negatively regulate the transforming growth factor β (TGF-β) family signaling pathway, particularly BMP signaling (Goto et al., 2007). Protein degradation by the ubiquitin-proteasome pathway plays a vital role in monitoring the abundance of many regulatory proteins. SMAD6 inhibits BMP signaling through reduced phosphorylation of SMAD2 and SMAD5, via competition with SMAD4, and through downregulation of SMAD4 with SMURF1 (Imamura et al., 1997; Hata et al., 1998; Moren et al., 2005). In addition to its conventional role as a negative regulator of TGF-β/BMP signaling, SMAD6 negatively regulates TLR-4 signaling through several mechanisms (Choi et al., 2006). In macrophages, SMAD6 induces MyD88 degradation by mediating the recruitment of SMURF proteins, which have E3-ubiquitin ligase activity (Lee et al., 2011). These previous studies led us to investigate if similar SMAD6-dependent negative feedback loop mechanisms would be applicable for the regulation of hepcidin, particularly mechanisms involving proteolytic-dependent degradation induced by BMP6 and Activin B, which are specifically relevant to the BMP/SMAD pathway (Andriopoulos et al., 2009; Besson-Fournier et al., 2012). Indeed, we show that both BMP6 and Activin B induced the ubiquitination and degradation of MyD88 and that this negative feedback loop is relevant to the modulation of hepcidin production in hepatocytes. Our results reveal for the first time, a potential mechanism of hepcidin negative feedback loop that involves ubiquitin-proteolytic degradation of modulatory proteins.

Our identification of the TIR domain of MyD88 as essential for the MyD88-SMAD4 interaction led to the question of the possible relevance of MyD88 mutations in the regulation of hepcidin expression and its consequent impact on cellular iron metabolism. However, genomic MyD88 mutations in humans are extremely rare, with approximately two dozen patients from six different countries identified thus far (Picard et al., 2011). In contrast, somatic MyD88 mutations, particularly the L265P missense substitution (Rossi, 2014), are frequent in a variety of mature B cell tumors. The most striking incidences are seen in Waldenström macroglobulinemia (WM) (Treon et al., 2012) where the mutation is detectable in more than 90% of patients (Gertz, 2015). There is increasing evidence that overactivation of TIR domain-mediated signaling is involved in inflammatory diseases and cancer growth (Ota et al., 2012). In B cell tumors, mutant MyD88 consistently results in a gain-of-function, leading to the activation of TLR downstream signaling pathways in the absence of cognate ligands and, ultimately, the elevation of NF-κB activity. In turn, this results in increased proliferation and survival of tumor cells (Ngo et al., 2011; Treon et al., 2012; Ansell et al., 2014). We therefore tested whether this mutation would affect hepcidin activation since the L265P mutation is located in the same TIR domain that is essential for the MyD88-SMAD4 interaction. We show that, in B cell lines, the L265P mutation of MyD88 can still bind to SMAD4 and results in enhanced SMAD4 expression to similar levels as seen with Wt MyD88. Furthermore, the gain-of-function of the L265P MyD88 is manifested by enhanced hepcidin expression and consequent iron accumulation in B cells. As a direct consequence of hepcidin activation, the expression of the iron exporter Ferroportin 1 (Nemeth et al., 2004) was significantly reduced in B cells that overexpressed the MyD88 L265P mutant, resulting in iron accumulation in these cells.

Importantly, previous studies report that hepcidin is produced by peripheral blood B cells from patients with WM and further show that hepcidin expression levels in B cells are also higher in WM patients compared to healthy donors (Ciccarelli et al., 2015). While hepcidin has been identified as an unequivocal contributor to anemia in WM, its production by B cells and monocytes is significantly lower than hepatocytes [data not shown and (Zhang and Rovin, 2010)]. However, as shown for monocytes (Theurl et al., 2008) and lymphocytes in general (Pinto et al., 2010), hepcidin produced by these cells may act as an autocrine regulator of iron accumulation. Accordingly, we show that B cell lines transfected with the MyD88 L265P mutant accumulated more iron when compared to wild-type MyD88. Our results therefore suggest a novel mechanism by which gain-of-function somatic MyD88 mutations may interfere with iron re-utilization by sequestrating iron and hence, contribute to anemia in WM (Weiss and Goodnough, 2005; Treon et al., 2013). This may help explain why, in many WM patients, anemia is of a severity out of proportion to bone marrow disease involvement (Treon, 2015). In addition, since iron is essential for cell survival, particularly for highly active cells such as tumor cells, increased hepcidin expression leading to higher cellular iron availability may further fuel tumor growth. In fact, there is increasing evidence that tumor cells manipulate hepcidin expression and regulation to meet their metabolic needs (Vela and Vela-Gaxha, 2018).

In summary, we report a new interaction between MyD88 and SMAD4 proteins that affects hepcidin induction though the BMP6/SMAD4 signaling pathway. Together, our data identify MyD88 as a potential player molecule in the BMP signaling pathway mediated by SMAD proteins. These findings may contribute to the identification of pathways and interacting elements that will provide further insight into the cross-regulation between iron metabolism and the immune system (Reuben et al., 2017).

## Author Contributions

MS-M and AC contributed to the investigation, validation, methodology, and formal analysis. FAM provided technical input regarding ubiquitination experiments and data analysis. MS-M and MMS additionally contributed to the conceptualization and writing of the original draft of the manuscript. MMS additionally contributed to the visualization, supervision, and funding acquisition of the study.

## Conflict of Interest Statement

The authors declare that the research was conducted in the absence of any commercial or financial relationships that could be construed as a potential conflict of interest.
