# Supplementary material for: MyD88 Regulates the Expression of SMAD4 and the Iron Regulatory Hormone Hepcidin
Source: Front Cell Dev Biol. 2018 Aug 31;6:105. doi: 10.3389/fcell.2018.00105 (PMC6127602; doi:10.3389/fcell.2018.00105)
Supplement: Supplementary file 1 [file Data_Sheet_1.PDF]

Supplementary figure 1

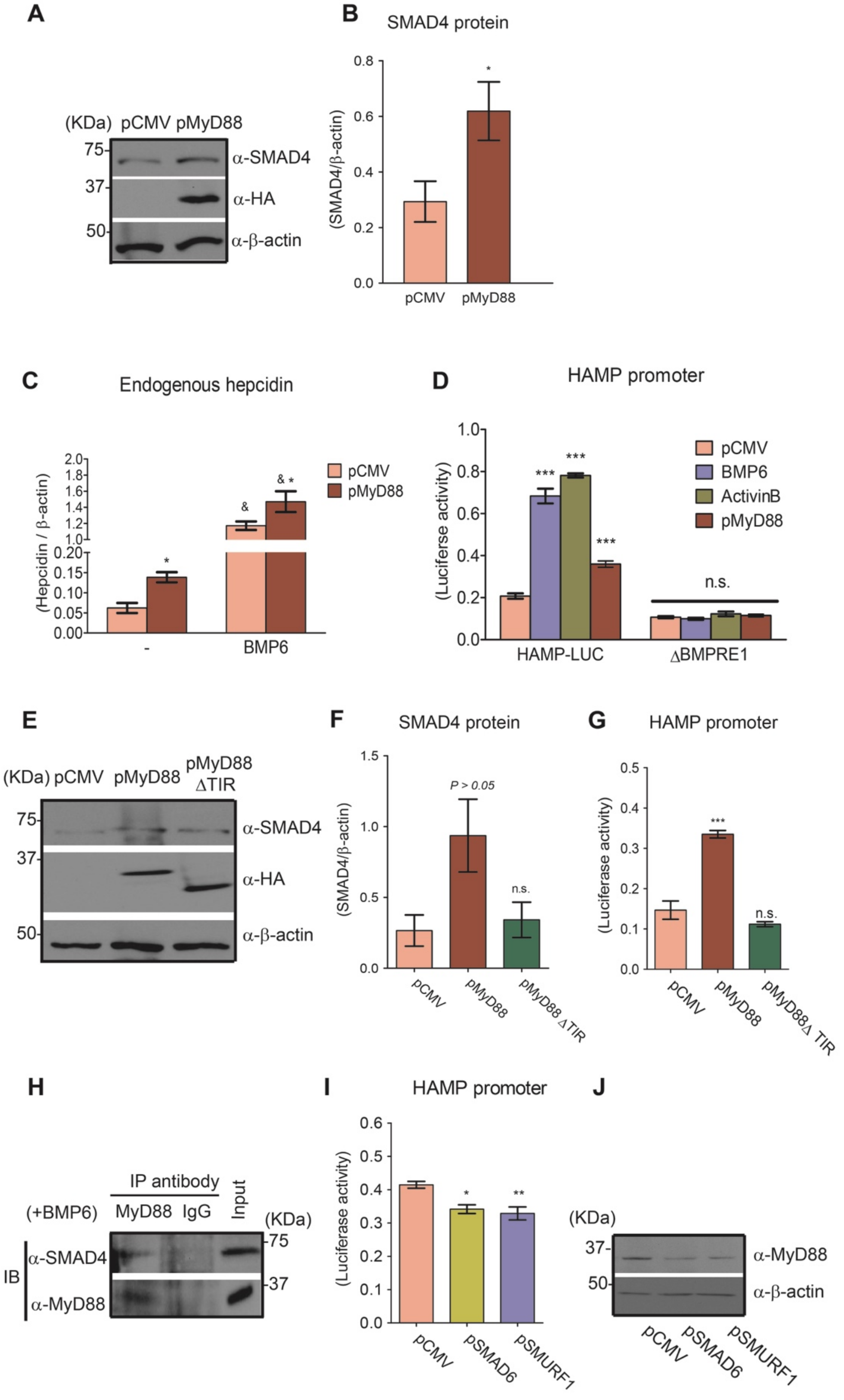

Supplementary figure 1. (A-D) MyD88 overexpression enhances SMAD4 and hepcidin expression. HepG2 cells were transiently transfected with an empty vector (pCMV) or HA-tagged MyD88 plasmid (pMyD88). (**A**) Total cell lysates were analyzed by western blotting for endogenous SMAD4 expression. Expression of the β-actin protein was used as a loading control. (**B**) Densitometric quantification of SMAD4 levels in western blots from three independent experiments. Results are presented as mean ± SEM. Statistical analyses were performed with Student's *t*-test. *$P < 0.01$ compared with empty vector (pCMV) (**C**) HepG2 cells were transiently transfected with an empty vector (pCMV) or HA-tagged MyD88 plasmid (pMyD88) and treated without (-) or with BMP6. Hepcidin (*HAMP)* mRNA levels were assessed by RT-PCR. Results are presented as mean ± SEM. *$P < 0.01$ compared with empty vector (pCMV) transfected cells. (**D**) **Mutation of the BMP-RE1 in the HAMP promoter abolishes HAMP-Luc induction by MyD88**. HepG2 cells were transiently co-transfected with HAMP-Luc or mutated HAMP-LucΔBMP-RE1 along with phRL-TK (*Renilla* Luciferase) as an internal control, and MyD88 plasmids (pCMV or MyD88). BMP6 and Activin B treatments were used as controls. Luciferase activity was assessed 24 hr after transfection. Results are presented as mean ± SEM of the relative activity (*Firefly*/*Renilla* ratio). ***$P < 0.0001$, and n.s. = not significant compared with empty plasmid (pCMV). **(E-G) Defective MyD88 mutant (ΔTIR domain) abolishes the induction of endogenous SMAD4 and HAMP promoter activation by MyD88 overexpression.** HepG2 cells were transfected with HAMP-Luc and pCMV or HA-tagged MyD88 vector (pMyD88) or the MyD88 vector lacking the TIR domain (pMyD88ΔTIR). **(E)** Expression of endogenous SMAD4 and transfected HA-tagged MyD88 was analyzed by western blotting. β-actin protein was used as a loading control. **(F)** Densitometric quantification of SMAD4 levels in western blots from three independent experiments. **(G)** Luciferase activity assessed 24 hr after transfection. Data are representative of a minimum of three experiments. Results are presented as mean ± SEM of the relative activity (*Firefly*/*Renilla* ratio). Statistical analysis was performed with one-way ANOVA for (**F**) and (**G**). ***$P < 0.0001$ and n.s. = not significant compared to pCMV. (**H**) Co-immunoprecipitation of endogenous MyD88 with endogenous SMAD4. HepG2 cells were treated with (+BMP6) for 24 hr. Cell lysates were subjected to immunoprecipitation (IP) with the anti-MyD88 or normal IgG antibody (as a control) and analyzed by immunoblotting (IB) with SMAD4 and MyD88 antibodies. (**I**) HepG2 cells were transiently co-transfected with HAMP-Luc in combination with empty plasmid (pCMV), pSMAD6, or pSMURF1. Luciferase activity was assessed 24 hr after transfection. Results are presented as mean ± SEM of the relative activity (*Firefly*/*Renilla* ratio). Statistical analysis was performed with one-way ANOVA. *$P < 0.05$, **$P < 0.001$. (**J**) Total cell lysates were analyzed for endogenous MyD88 by immunoblotting using an anti-MyD88 antibody. Expression of the β-actin protein was used as a loading control. All data are representative of at least three independent experiments.

Supplementary figure 2

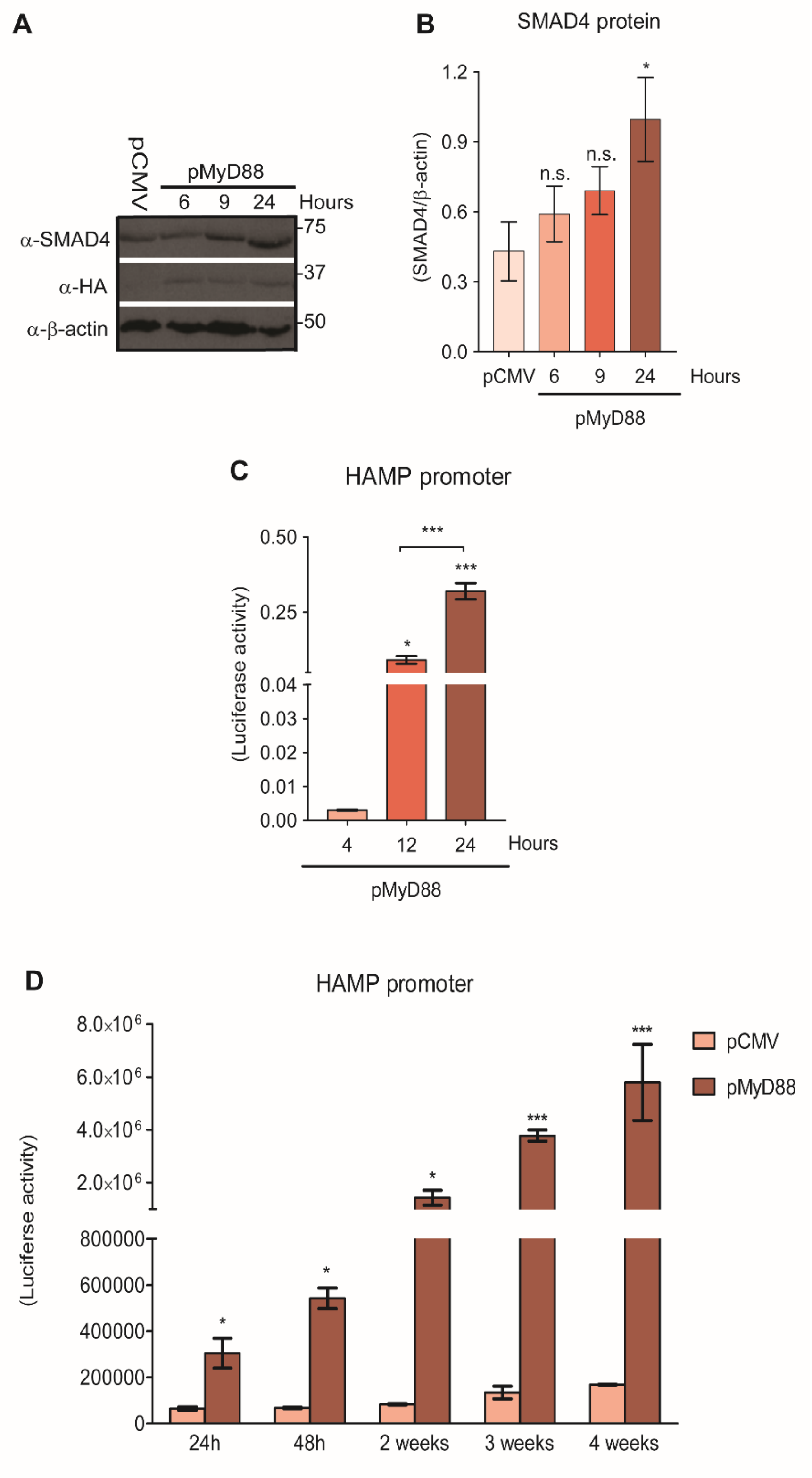

Supplementary figure 2. **MyD88 expression influences SMAD4 levels in Huh7 hepatoma cells.** (**A**) Huh7 cells were transiently transfected with HA-tagged MyD88 plasmid (pMyD88) for 6, 9 and 24 hr or were transfected with the empty plasmid pCMV as negative control. Total cell lysates were analyzed by western blotting for endogenous SMAD4 expression. Expression of the β-actin protein was used as a loading control. (**B**) Densitometric quantification of SMAD4 levels in western blots from three independent experiments. Results are presented as mean ± SEM. *$P < 0.05$*, and n.s. = not significant compared with empty plasmid (pCMV). (**C**) Huh7 cells were transiently co-transfected with HAMP-Luc along with phRL-TK (*Renilla* Luciferase) as an internal control, and pMyD88 plasmids. Luciferase activity was assessed after 4, 12 and 24 hr after transfection. Results are presented as mean ± SEM of the relative activity (*Firefly*/*Renilla* ratio). *$P < 0.05$ and ***$P < 0.0001$ compared with empty plasmid (pCMV). The results are representative of at least three independent experiments. Statistical analysis was performed with one-way ANOVA. (**D**) Huh7 cell lines were stably co-transfected with HAMP-Metluc2 along with pCMV or pMyD88. Cells were grown under selection using G418 (200 µg/ml), which was added in the culture medium 24 hours post-transfection. Luciferase activity was measured at 24 hr, 48 hr, 2 weeks, 3 weeks and 4 weeks after transfection. Results are presented as mean ± SEM of the relative activity (*Firefly*/*Renilla* ratio). *$P < 0.05$ and ***$P < 0.0001$ compared with empty plasmid (pCMV). Statistical analysis was performed with two-way ANOVA.

Supplementary figure 3

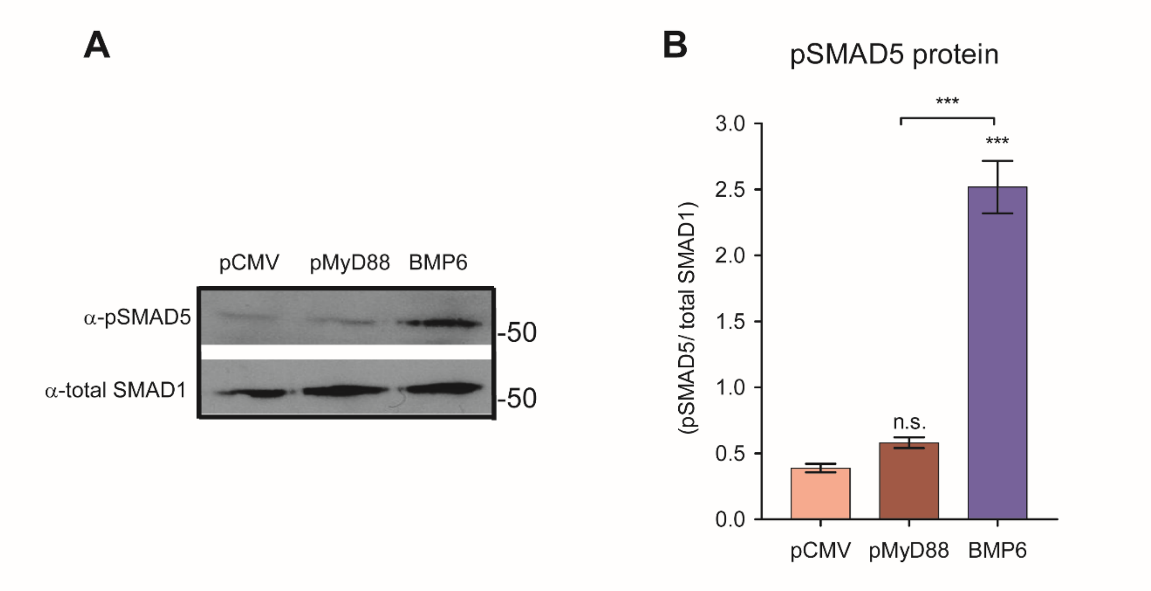

Supplementary figure 3. (**A**) Huh7 cells were transiently transfected with an empty vector (pCMV) or HA-tagged MyD88 plasmid (pMyD88) or with pMyD88 or treated with BMP6 (BMP6) as a positive control. Total cell lysates were analyzed by western blotting for endogenous phosphorylated SMAD5 (pSMAD5) and total SMAD1. (**B**) Densitometric quantification of pSMAD5 levels in western blots from three independent experiments. Statistical analysis was performed with one-way ANOVA. Results are presented as mean ± SEM. ***$P < 0.0001$ and n.s. = not significant compared with empty plasmid (pCMV).

Supplementary figure 4

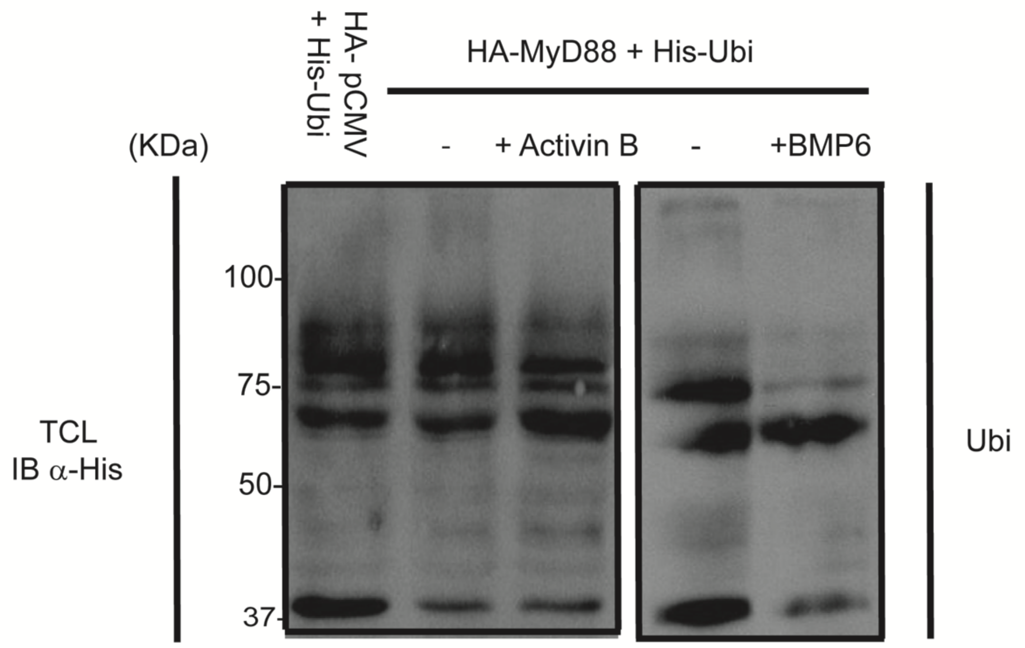

Supplementary figure 4: Huh7 cells were co-transfected with HA-tagged MyD88 and His-tagged Ubiquitin (His-Ubi), and treated with BMP6 or Activin B. Total cell lysates (TCL) were immunoblotted with anti-His (Ubi) antibody as a control for Figure 4A.

Supplementary figure 5

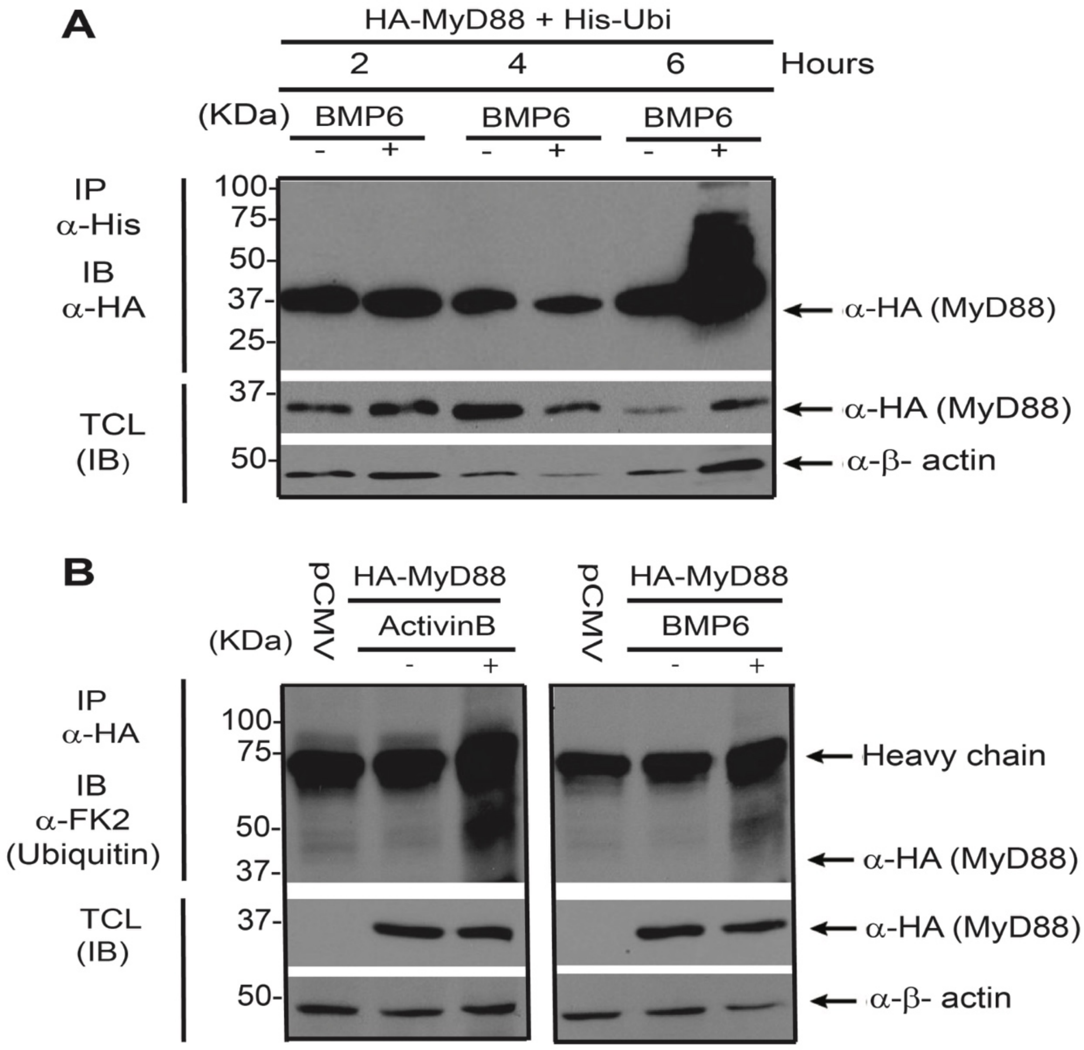

Supplementary figure 5. (A) Huh7 cells were co-transfected with HA-tagged MyD88 and His-tagged Ubiquitin (His-Ubi) for 24 hr prior to BMP6 treatment (25 ng/ml) for 2, 4 and 6 hours. HA-MyD88 ubiquitination was examined by immunoprecipitation (IP) using anti-His antibody, followed by immunoblotting (IB) with anti-HA antibody. Total cell lysates (TCL) before IP were immunoblotted with anti-HA and anti-β-actin antibodies. **(B)** Huh 7 cells were transfected with the empty plasmid pCMV or HA-tagged MyD88 for 24 hr and treated with Activin B or BMP6 for 6 hr. Endogenous ubiquitination was examined by immunoprecipitation (IP) using anti-HA followed by IB with anti-FK2 (ubiquitin).

Supplementary figure 6

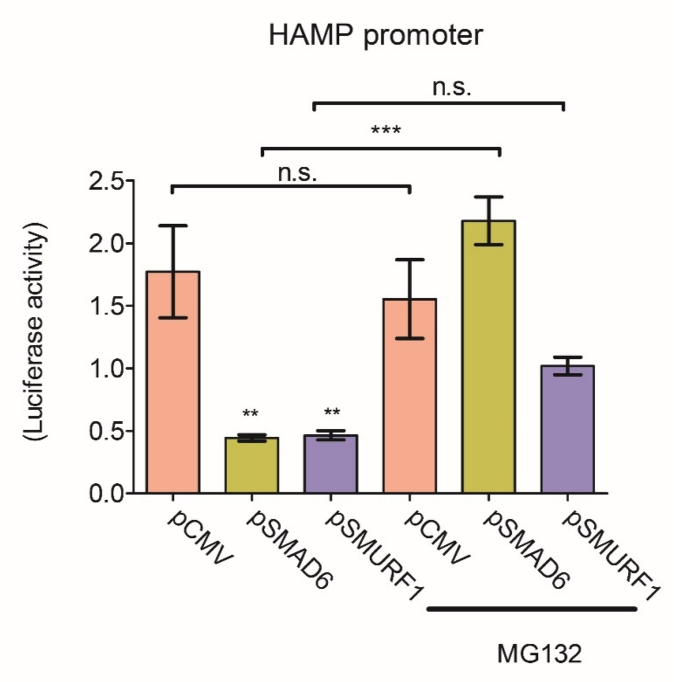

Supplementary figure 6. Huh7 cells were co-transfected with HAMP-luc and phRL-TK (Renilla Luciferase) along with pCMV, pSMAD6 or pSMURF1. After 24 hr, cells were treated with MG132 (10 mM) for 4 hours. Luciferase activity was assessed and results are presented as mean ± SEM of the relative activity (*Firefly*/*Renilla* ratio). Statistical analysis was performed with one-way ANOVA. Results are presented as mean ± SEM. $^{**}P < 0.001$, $^{***}P < 0.0001$ and n.s. = not significant compared with empty plasmid (pCMV).

Supplementary figure 7

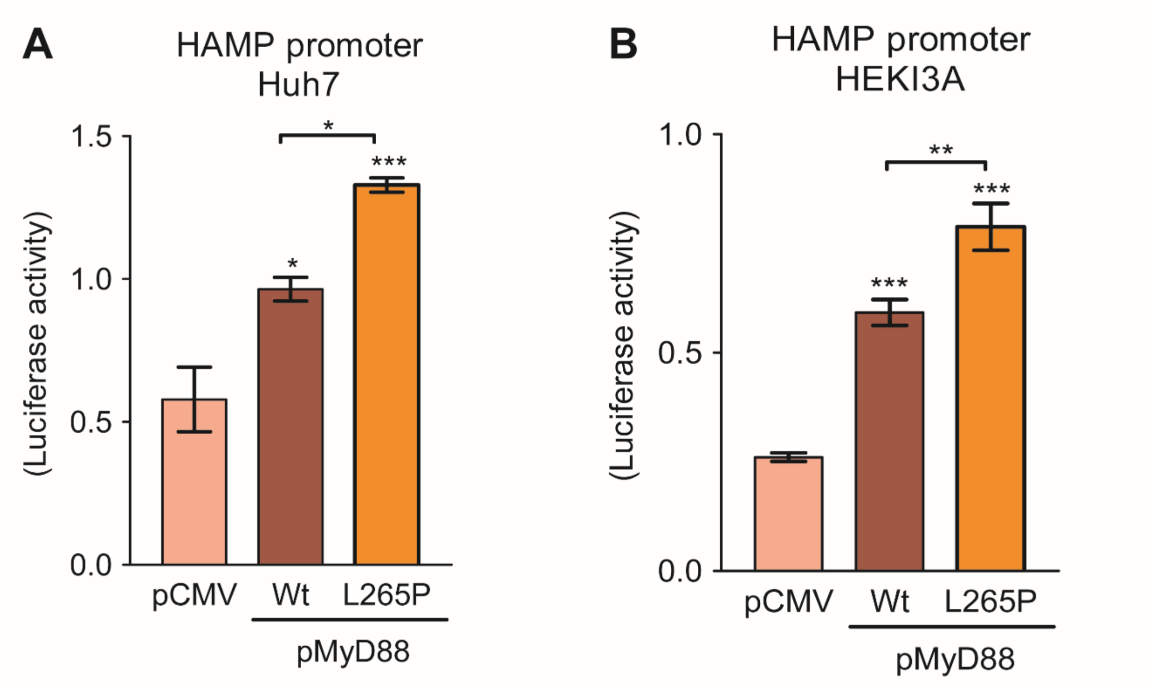

Supplementary figure 7. (**A**) Huh7 and (**B**) HEKI3A cells were co-transfected with HAMP-Luc and control (pCMV) or MyD88 plasmids (pMyD88): wild-type (Wt) or mutated MyD88 (L265P). Luciferase activity was assessed 24 hr after transfection. Results are presented as mean ± SEM. **P < 0.05, **P < 0.001*, ****P < 0.0001*.
